
JOURNAL INFORMATION
==============================
NLM Title Abbreviation: ScientificWorldJournal
Journal ID: ScientificWorldJournal
NLM Journal ID: 101131163
Journal ID: TSWJ

The Scientific World Journal

ISSN: 2356-6140
EISSN: 1537-744X
Publisher: Wiley

ARTICLE INFORMATION
==============================
PMCID: PMC5720115
PMID: 21258755
DOI: 10.1100/tsw.2011.18
Article ID: 202369
Article version: 1
Subjects: Clinical Image

Intracavitary and Endobronchial Tuberculosis

Alsumrain Mohammad 1 2 *
Alberaqdar Enis 1 2
Bennett-Vernner Arianne 1 2
Klukowicz Alan 1 2
Miller Richard 1 2
1 Pulmonary Department, Saint Michael's Medical Center, Newark, NJ, USA
2 Pulmonary and Critical Care Fellowship Program, School of Health and Medical Sciences, Seton Hall University, South Orange, NJ, USA
*Mohammad Alsumrain: alsumrain@yahoo.comAcademic Editor: Daniel Batlle

Publication date: 2011
Electronic publication date: 2011 Jan 18
Volume: 11
First page: 130
Last page: 132
Received 2010 Oct 4; Revised 2010 Dec 3; Accepted 2010 Dec 14
Copyright: Copyright © 2011 Mohammad Alsumrain et al.
Copyright year: 2011
License: This is an open access article distributed under the Creative Commons Attribution License, which permits unrestricted use, distribution, and reproduction in any medium, provided the original work is properly cited.
License URL: https://creativecommons.org/licenses/by/3.0/

Keywords: tuberculosis, cavitations, bronchoscopy

==============================
Clinical Image
*TheScientificWorldJOURNAL* (2011) 11, 130–132
ISSN 1537-744X; DOI 10.1100/tsw.2011.18

TheScientificWorldJOURNAL
www.thescientificworld.com

### Intracavitary and Endobronchial Tuberculosis

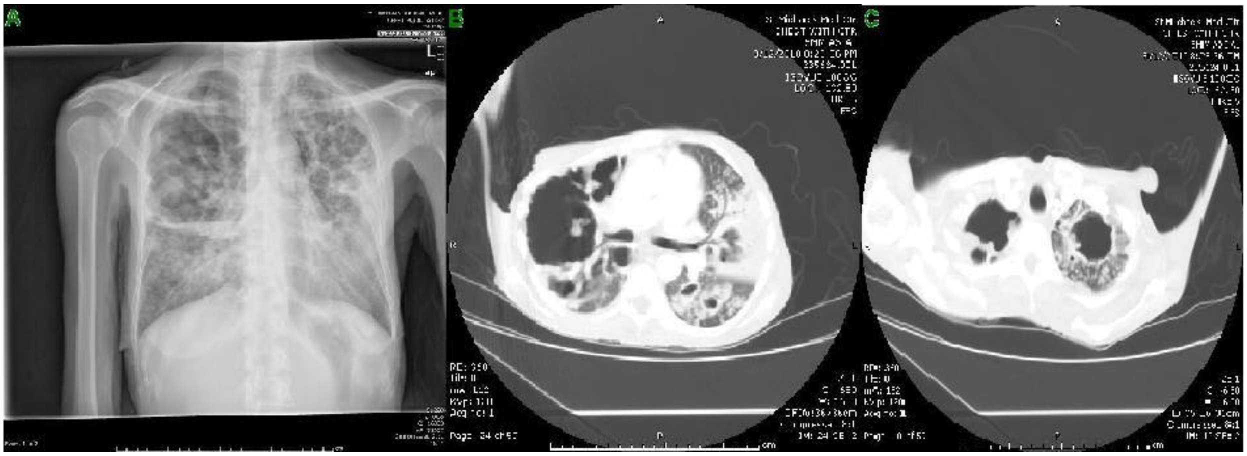

**FIGURE 1.** (A) Chest X-ray of the patient showing bilateral cystic and bullous infiltrates, and (B, C) CT scans of the chest showing a large cavitary lesion in the right upper lobe.

Mohammad Alsumrain*, Enis Alberaqdar, Arianne Bennett-Venner, Alan Klukowicz, and Richard Miller

*Pulmonary Department, Saint Michael's Medical Center, Newark, NJ; Pulmonary and Critical Care Fellowship Program, School of Health and Medical Sciences, Seton Hall University, South Orange, NJ*

**E-mail**: alsumrain@yahoo.com

*Received October 4, 2010; Revised December 3, 2010; Accepted December 14, 2010; Published January 18, 2011*

**KEYWORDS**: tuberculosis, cavitations, bronchoscopy

Flexible bronchoscopy is a widely accepted diagnostic modality for tuberculosis[1,2,3], specifically in patients who cannot produce an adequate sputum sample for an acid fast bacilli (AFB) smear in a reasonable period of time despite hypertonic saline induction. Bronchial aspirates obtained during bronchoscopy considerably increase the diagnostic yield in tuberculosis[4].

We present the case of an interesting and unusual bronchoscopic finding in a 46-year-old African American woman who had been homeless for 10 years and resided in an inner city train station. She had no significant past medical history and had not been on any medications. The patient presented with a complaint of cough with greenish sputum, but no hemoptysis, for 5 months. She had a noted weight loss of 90 lb over a 1-year period and she also complained of shortness of breath on exertion. She denied fever, chills, night sweats, or chest pain. On physical examination, the patient was cachectic; she looked

*Corresponding author.
©2011 with author.
Published by TheScientificWorld; www.thescientificworld.com older than her stated age, pale, with poor oral hygiene, and multiple aphthous ulcers on the tongue. Chest examination revealed good air entry with bilateral crackles. The patient was admitted to a respiratory isolation room, and chest X-ray showed bilateral cystic and bullous interstitial infiltrates (Fig. 1, panel A). CT scan of the chest revealed diffuse cystic bronchectasis, cavitary and nodular infiltrates, and a large cavitary lesion in the right upper lobe (Fig. 1, panels B and C). A PPD skin test was positive and greater than 15 mm. The patient was unable to produce an adequate sputum sample for an AFB smear, even after trials of sputum induction with hypertonic saline. Therefore, bronchoscopy was performed to obtain bronchoalveolar lavage fluid and bronchial brushings. Endobronchially, the airway demonstrated that the anterior segment of the right upper lobe bronchus was contiguous with an opening to a right-sided cavity (Fig. 2, panel A), which was lined with inflammatory and necrotic material (Fig. 2, panels B and C). The areas were brushed and lavaged for culture and cytology. There were patchy whitish lesions noted diffusely on the tracheal wall (Fig. 2, panel D), which was also brushed and cultured. AFB smears were strongly positive from the bronchial washing. The patient was started on four-drug therapy for tuberculosis.

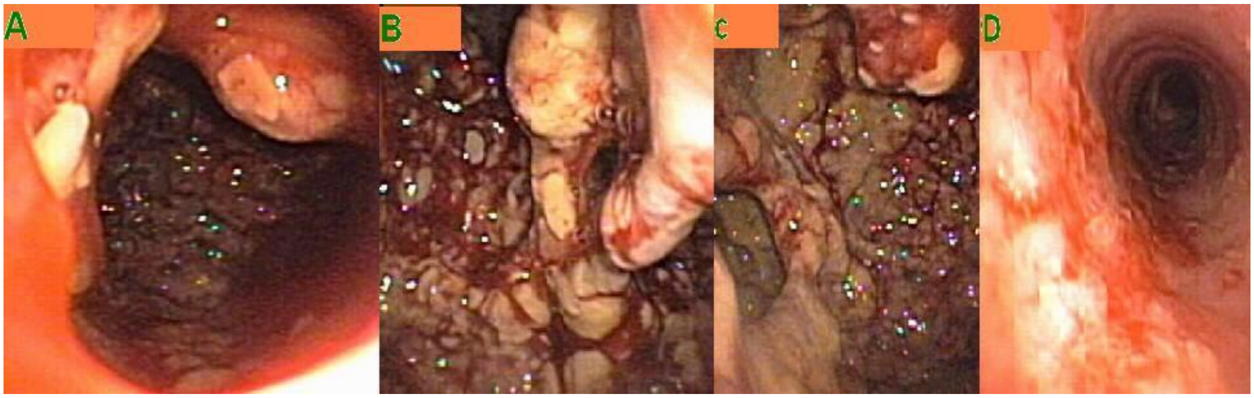

**FIGURE 2.** Bronchoscopic views showing (A) the entrance of the anterior segment of the right upper lobe of the lung contiguous with an opening to a right-sided cavity, (B, C) necrotic material inside the cavity in the right upper lobe, and (D) white plaques in the tracheal wall.

The bronchoscopic findings in endobronchial tuberculosis are variable. They can include actively caseating, edematous-hyperemic, fibrostenotic, tumorous, granular, ulcerative, and nonspecifically bronchitic lesions[5]. Although we were able to visualize the ulcerative changes in the trachea, there were no other obvious endobronchial lesions. Our research did not reveal any available literature describing the findings of endoscopically observed intracavitary tuberculosis. The dramatic appearance of the intracavitary disease was remarkable, and the fortuitous finding of the bronchial connection to the large cavity was incidental since there was no indication of direct connection on any of the radiographic techniques.

### REFERENCES

1. Willcox, P.A., Benatar, S.R., and Potgieter, P.D. (1982) Use of the flexible fibreoptic bronchoscope in diagnosis of sputum-negative pulmonary tuberculosis. *Thorax* **37(8),** 598–601.
2. Danek, S.J. and Bower, J.S. (1979) Diagnosis of pulmonary tuberculosis by flexible fiberoptic bronchoscopy. *Am. Rev. Respir. Dis.* **119(4),** 677–679.
3. Uddenfeldt, M. and Lundgren, R. (1981) Flexible fiberoptic bronchoscopy in the diagnosis of pulmonary tuberculosis. *Tubercle* **62(3),** 197–199.

4. Schoch, O.D., Rieder, P., Tueller, C., Altpeter, E., Zellweger, J.-P., Rieder, H.L., Krause, M., and Thurnheer, R. (2007) Diagnostic yield of sputum, induced sputum, and bronchoscopy after radiologic tuberculosis screening. *Am. J. Respir. Crit. Care Med*. **175(1),** 80–86.
5. Chung, H.S. and Lee, J.H. (2000) Bronchoscopic assessment of the evolution of endobronchial tuberculosis. *Chest* **117(2),** 385–392.

**This article should be cited as follows:**

Alsumrain, M., Alberaqdar, E., Bennett-Venner, A., Klucowicz, A., and Miller, R. (2011) Intracavitary and endobronchial tuberculosis. *TheScientificWorldJOURNAL* **11**, 130–132. DOI 10.1100/tsw.2011.247.
